# Macro-scale transport of the excitation energy along a metal nanotrack: exciton-plasmon energy transfer mechanism

**DOI:** 10.1038/s41598-018-36627-2

**Published:** 2019-01-14

**Authors:** Igor Khmelinskii, Serguei N. Skatchkov, Vladimir I. Makarov

**Affiliations:** 1 University of the Algarve, FCT, DQF and CEOT, 8005-139 Faro, Portugal; 2 Universidad Central del Caribe, Bayamón, PR 00960-6032 USA; 3 University of Puerto Rico, Rio Piedras Campus, PO Box 23343, San Juan, PR 00931-3343 USA

## Abstract

Presently we report (i) excited state (exciton) propagation in a metal nanotrack over macroscopic distances, along with (ii) energy transfer from the nanotrack to adsorbed dye molecules. We measured the rates of both of these processes. We concluded that the effective speed of exciton propagation along the nanotrack is about 8 × 10^7^ cm/s, much lower than the surface plasmon propagation speed of 1.4 × 10^10^ cm/s. We report that the transmitted energy yield depends on the nanotrack length, with the energy emitted from the surface much lower than the transmitted energy, i.e. the excited nanotrack mainly emits in its end zone. Our model thus assumes that the limiting step in the exciton propagation is the energy transfer between the originally prepared excitons and surface plasmons, with the rate constant of about 5.7 × 10^7^ s^−1^. We also conclude that the energy transfer between the nanotrack and the adsorbed dye is limited by the excited-state lifetime in the nanotrack. Indeed, the measured characteristic buildup time of the dye emission is much longer than the characteristic energy transfer time to the dye of 81 ns, and thus must be determined by the excited state lifetime in the nanotrack. Indeed, the latter is very close to the characteristic buildup time of the dye emission. The data obtained are novel and very promising for a broad range of future applications.

## Introduction

Extensive studies of quantum confinement (QC) in nanostructures of different nature and topology have begun over 110 years ago^1^. When one or more of the characteristic dimensions of a physical object become smaller than the electron de Broglie wavelength in the respective material, QC occurs with discrete levels appearing in the electronic energy spectrum of the system. Such effects are very well known in quantum dots^2–5^, graphene quantum disks^6–11^, nanotubes and nanowires^12–15^, metal and semiconductor thin films^16–21^, and other systems. Detailed studies of QC in different systems are of fundamental significance, improving our understanding of the structure of matter, with very promising technological applications in microelectronics, optoelectronics, solar light harvesting, surface coatings, and nanofabrication. Very interesting results were reported earlier^22,23^, demonstrating macroscopic energy propagation along metal nanotracks, interpreted as a consequence of QC in the nanotrack. Additionally, light energy transfer along nanostructured waveguides was also studied, with the waveguides constructed of a metal nanolayer deposited on the surface of a nanostructured dielectric^19–21^, with the long-distance light energy transfer interpreted in terms of quasi-classical plasmon/polaron theory. In this latter case, however, QC has to be considered in conjunction with the plasmon/polaron theory, as the longitudinal plasmon wave interacts with transversal waves generated by electronic oscillations in the direction normal to the waveguide axis, appearing due to QC. Analyzing plasmon dynamics in the bulk metal, we may easily neglect the effects of transverse electron waves, with longitudinal electron waves determining the system properties^24^. However, when the sample thickness is reduced to the nanometer scale, both electron waves become important for an adequate description of the electronic gas dynamics, therefore transversal waves must also be included. Similar effects are very clearly observed in nanodots^2–5^, also having discrete electronic energy spectra.

As we already noted, one-dimensional QC is observable in uniform metal nanotracks and nanolayers, where both absorption and emission spectra have discrete structure^16–19,22,23^. QC was reported earlier in metallic thin films^16–19^, including spectroscopic characteristics of Au, Fe, Co, Ni and Cr thin films. Similar effects were reported for SnO_2_ and Si semiconductor nanofilms and sandwich assemblies that included semiconductor and metal nanofilms^20,21,25^. QC in these films was interpreted using a simple model^16–21,25^ that describes electrons in a one-dimensional box with infinite potential walls^26^, with the steady-state energies given by the expression:

1 $${E}_{n}=\frac{{\pi }^{2}{h}^{2}{n}^{2}}{2{m}_{eff}}{a}^{2}$$

where *m*_*eff*_ is the effective electron mass in the respective material, with the following values measured in different materials; Au: 0.93, Fe: 0.027, Co: 0.17, Ni: 0.13, Si: 0.17, SnO_2_: 0.21 and Cr: 0.047 (all reported as fractions of the free electron mass). There is a good understanding of the spectral data of such nanolayers, however, no detailed study and/or theoretic analysis of the energy propagation along metal nanotracks has ever been reported. Indeed, energy propagation was only probed for a Cr nanotrack^22,23^. We believe that a more detailed investigation of the energy propagation will create a better understanding of the excited state dynamics in nanostructured systems, with possible practical outcomes for the fabrication of novel optoelectronic devices. We must also note that the simple models used until now do not describe the excited-state wave package propagation along the nanostructure, thus, a more detailed theoretical approach is required.

Another important aspect that may be used to probe the excited state dynamics in nanofilms is their interaction with other energy acceptors, e.g. adsorbed dyes. At this time we are unaware of any publications reporting such studies. The respective results should impact the analysis of photomicrographs of biological samples, e.g. of proteins tagged by dye molecules, with both the biomolecule and the dye simultaneously excited by an external light source. Additionally, the studies of energy transfer may impact the solar light harvesting applications. Indeed, we may use nanofilms to absorb photons, with the absorbed photon energy subsequently transferred along the film to the locations where it is utilized e.g. for producing electric current^27–30^.

Presently, we studied dynamics of the energy transfer from a Co nanotrack 11.421 nm thick excited at 630 nm to oxazine-170 dye that has an absorption spectrum peaking at around 630 nm and the emission spectrum at 660–728 nm. We report that excitation of the Co nanotrack generates dye emission, due mostly to the dye adsorbed by the nanotrack. Simultaneously, the excited-state nanotrack also emits its own characteristic emission, with the spectrum different from that of the dye. We compare spectroscopic and kinetic data obtained for the nanotrack with the adsorbed dye to those recorded for a Co nanolayer with the same thickness. We also report a detailed theoretical analysis of the presently obtained results. Our novel findings may be used to explain the mechanism of light propagation along the intermediate filaments that are packed inside retinal Müller cells^22,23,31,32^ and conduct light against the laws of classic optics^22,23,31,32^. Finally, our results are very promising for a wide range of future applications.

## Experimental

CaF_2_ and AlN substrates sized 25 × 12.5 mm^2^ and 1 mm thick (Esco Optics Inc. and Hexo Technology Inc.) were used to deposit the samples. Commercial Co targets (Sigma/Aldrich) were used to deposit nanocrystalline films using a commercial sputtering/thermo-evaporation Benchtop Turbo deposition system (Denton Vacuum). Co nanolayers/nanotracks were deposited using sputtering, with the substrate at 475 °C in all experiments. The deposited films were annealed for 2 hours at 900 °C in pure nitrogen gas at atmospheric pressure. The film thickness was controlled by XRD^33^, on an XPert MRD system (PANalytic), calibrated using standard nanofilms of the same material. The estimated absolute uncertainty of film thickness was 0.2%; the relative uncertainties were much smaller, determined by the shutter opening times of the deposition system.

Commercial oxazin-170 dye (Coherent Inc.) and high purity methanol (Sigma-Aldrich; 99.98wt%) were used without any additional purification.

**Figure 1:**
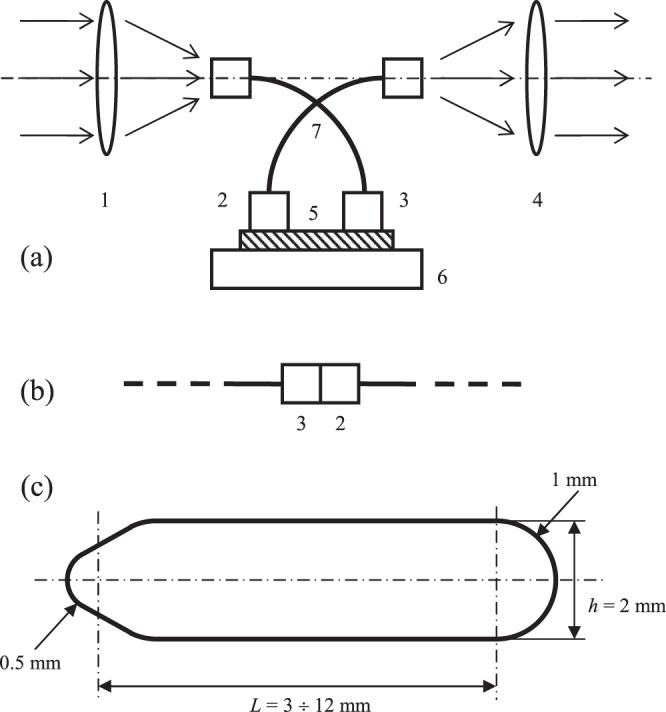
(**a**) Block diagram of the assembly used for measuring energy transfer along Co nanotracks sized (3 ÷ 12) × 2 mm^2^ and 11.421 nm thick, where 1 is a fused silica lens focusing probing radiation onto one connector 2 of a multimode fiber optic cable 7, 3 is the connector of the second fiber optic cable 7, 4 is a fused silica collimator lens providing a parallel beam, the latter passing first through a filter or going directly to the photodetector, 5 – Co nanotrack deposited on the AlN substrate 6; (**b**) the connectors of the two fiber cables 2 and 3 were connected to each other directly for recording the baseline; (**c**) the shape of the nanotrack.

Absorption and emission spectra were recorded on a Hitachi U-3900H UV-Visible Spectrophotometer and Edinburgh Instruments FS5 Spectrofluorometer. The absorption spectra in the mid- and near-IR were recorded on a PF2000 FTIR spectrometer (Perkin Elmer). The absorption spectra are presented as the difference of the transmission and the reflection spectra. The spectral peak maxima were precisely located using PeakFit software (Sigmaplot). The polynomials were fitted and the fitting uncertainties estimated using the LINEST function in Excel (Microsoft). Photo-induced response measurements were performed using a high-pressure Xe lamp (1000W, Ariel Corporation, Model 66023), a monochromator (Thermo Jarrell Ash, Mono Spec/50), a DET10A Biased Si detector (THORLABS, supplied with the spectral calibration curve), a model 2182A nanovoltmeter (Keithley Instruments) connected to a computer by a GPIB interface, and home-made software in the LabView programming environment (National Instruments). The light beam of the Xe lamp was filtered by interference filters with the pass-band at 542 nm, with the radiation exciting the Co nanolayer at normal incidence. We detected the emission of the nanotrack at its other end, as shown in Fig. 1. He-Ne laser (NewPort Inc.) generated light at 633 nm, and its output in the 0.5–35 mW power range was used as a narrow-band steady-state excitation source.

We recorded time-resolved emission using the fundamental harmonics of LPD-2000 (Λ-Physics) dye laser with Rhodamine-101 dye (with frequency multiplication on a BBO crystal; Λ-Physics) as the excitation source. Rhodamine-101 fundamental frequency was tuned in the 611–662 nm spectral range (frequency-doubled to obtain 305–331 nm radiation), pumped by the second harmonics of a YAG laser (532 nm, Surelight-II, Continuum Inc.). The laser pulse duration was about 7–10 ns. The dye laser radiation was defocused onto the entire free surface of the respective film. The emission was collected by a spherical 30-cm focal distance CaF_2_ lens and detected by a photodiode (PD1; DET10A Biased Si Detector from THORLABS) or a photomultiplier (Hamamatsu, Hamamatsu-PMT-H9305-03), after passing through a neutral density filter. The data acquisition system used a PC, a digital oscilloscope (WaveSurfer 400 series, LeCroy), two digital delay generators (DG-535, Stanford Research), a photo-detector (PD: DET10A Si Detector from THORLABS) or a photomultiplier (Hamamatsu, Hamamatsu-PMT-H9305-03), two boxcar integrators (SR-250, Stanford Research), a fast amplifier (SR-240, Stanford Research), and a computer interface board (SR-245 Stanford Research). The emission signal was monitored by the digital oscilloscope and averaged in all spectroscopic experiments, typically using 5 laser pulses per frequency step. The output energy was controlled and the monochromator scan operated using a PD and PCI-6034E DAQ I/O board (National Instruments), with the control code in the LABVIEW environment running on a second Dell PC. The presently used experimental methods produced 2.5 ns time resolution. The system used for the measurements of the energy transmission along the nanotrack is shown schematically in Fig. 1.

Each of the two fiber optic cables had its optical core 1 mm in diameter, connected to one of the ends of the Co nanotrack, with variable spacing between the axes of the cores. The fiber-interfaced sample nanotrack was thus reproducibly connected to the spectral equipment used in the measurements.

We measured the emission quantum yields using a calibrated radiation source (High Pressure Hg lamp: ESI 1200 100W MSR Lamp; Planet Bulb Inc.) and a calibrated photodiode (PD: DET10A Si Detector from THORLABS). All of the measurements were performed at 300 K.

## Results

### Absorption spectra

**Figure 2:**
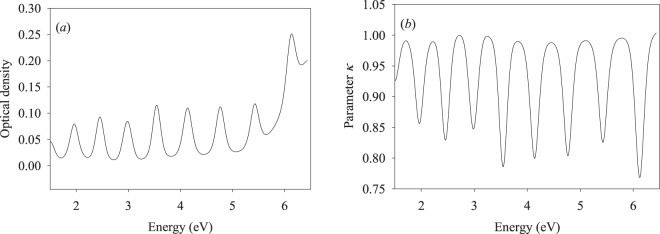
(**a**) Absorption spectrum of a 11.421 nm Co film deposited on CaF_2_ substrate; (**b**) energy transfer (transmission) spectrum of a 11.421 nm Co nanotrack.

Absorption spectrum of a Co nanolayer 11.421 nm thick on a CaF_2_ substrate is shown in Fig. 2a. This spectrum was recorded using the standard procedure with correction for the sample reflection and absorption of a clean CaF_2_ substrate. The spectrum describing the light energy transfer along the nanotrack (NT), which we shall refer to as the transmission spectrum of the nanotrack, was recorded using a commercial spectrometer and the fiber-optical interface of Fig. 1a, with the baseline recorded as in Fig. 1b subtracted. The resulting spectrum is shown in Fig. 2b.

In Fig. 2b  $\kappa =\frac{D(E)}{{D}_{\max }}$. Here *D*(*E*) is the transmission coefficient in function of the scanning light energy and *D*_*max*_ is the maximum value in the *D*(*E*) spectrum. The band maxima of the spectrum shown of Fig. 2a and the band minima of Fig. 2b are listed in Table 1.

**Table 1:** The band maxima of the absorption spectrum of Fig. 2a and the respective band minima of the spectrum of Fig. 2b.

Band number	Maximum, eV (Fig. 2a)	Minimum, eV (Fig. 2b)
1	1.9639	1.9639
2	2.4574	2.4573
3	2.9844	2.9845
4	3.5451	3.5450
5	4.1392	4.1392
6	4.7670	4.7670
7	5.4284	5.4283
8	6.1233	6.1233

**Figure 3:**
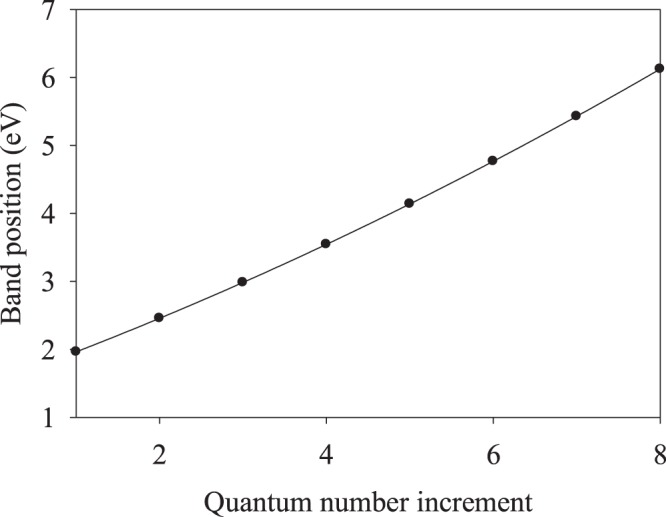
Band maxima/minima vs. the quantum number increment.

We conclude from Table 1 that the positions of the band maxima (Fig. 2a) and minima (Fig. 2b) are in excellent agreement between each other, with the respective data plotted in Fig. 3 in function of the quantum number increment.

Fitting the experimental data by the second-order polynomial of Eq. (1) produced the quantum number of the highest populated state (*n* = 9 for the electronic ground state of the absorption transitions) and the effective electron mass (*f* = 0.17123), which are in good agreement with the earlier reported values^22^. Thus, the electronic state structure in the Co nanotrack is the same as that in Co thin films. As follows from Fig. 2b, the excitation energy transfer efficiency along the nanotrack achieves the maximum value of 0.23 at 6.1233 eV excitation. In the following subsection, we shall discuss the dynamics of macro-scale energy transfer.

### Dynamics of energy transfer along the nanotrack

**Figure 4:**
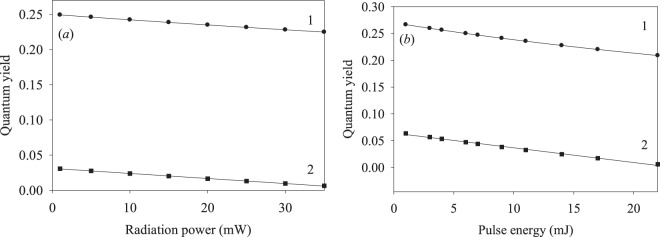
Efficiency (quantum yield) of the energy transfer along the nanotrack (circles) and the energy emitted by the nanotrack (squares): (**a**) steady-state excitation at 633 nm; (**b**) pulsed excitation at 630 nm.

All of the experiments on the energy transfer along the Co nanotrack used either steady-state excitation at 633 nm with the power adjustable in the 0.5–35 mW range, or pulsed excitation at 630 nm with the pulse energy adjustable in the 1–22 mJ/pulse range. Such excitation induces the *n* = 14 ← *n* = 9 electronic transition at ca. 1.9639 eV. The power dependence of the energy transfer efficiency upon steady-state excitation is shown in Fig. 4a. The laser pulse energy dependences of the transferred energy and energy emitted by the nanotrack are shown in Fig. 4b. The results of Fig. 4 are plotted against the baseline values recorded with the measurement scheme of Fig. 1a and b.

**Figure 5:**
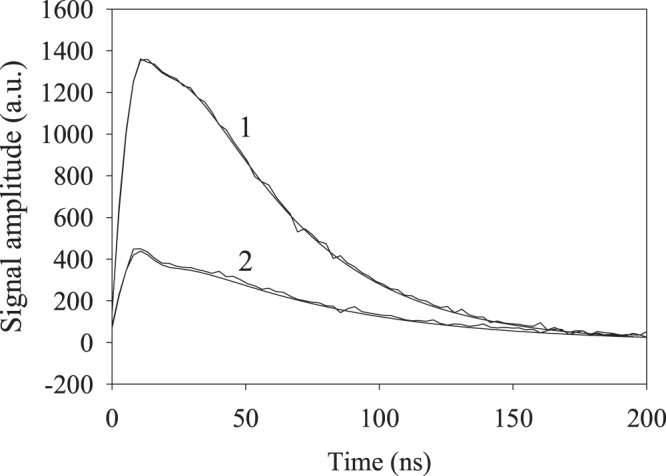
(1) Dynamics of the radiation transmitted along the nanotrack and (2) of the radiation emitted by the nanotrack; pulsed excitation at 630 nm.

It follows from Fig. 4 that the quantum yield of the energy transmission along the nanotrack is significantly higher than the emission quantum yield of the same nanotrack. Thus, our nanotrack is quite an efficient energy transfer system. Time-resolved experiments were carried out using pulsed excitation at 630 nm wavelength, with typical kinetic traces of the transmitted and emitted radiation shown in Fig. 5.

The kinetic traces were fitted using the function

2 $$I(t)={A}_{1}{e}^{-{(\frac{t-{t}_{0}}{{\tau }_{las}})}^{2}}+{B}_{1}({e}^{-\frac{t}{{\tau }_{1}}}-{e}^{-\frac{t}{{\tau }_{2}}})$$

where *A*_1_ and *B*_1_ are the amplitudes, *τ*_*las*_, *τ*_1_ and *τ*_2_ are the laser pulse duration, signal decay time and signal buildup time, respectively. The fitting parameters are listed in Table 2.

**Table 2:** Fitting parameters for the emission dynamics obtained using Eq. (2).

Parameter	*A*_1_, a.u.	*t*_0_, ns	*τ*_*las*_, ns	*B*_1_, a.u.	*τ*_1_, ns (decay)	*τ*_2_, ns (buildup)
Transmitted to the second light-guide	165 ± 6	7.7 ± 0.4	7.9 ± 0.5	3170 ± 62	41.7 ± 1.3	13.1 ± 0.8
Emitted by the nanotrack	173 ± 7	7.6 ± 0.5	8.1 ± 0.6	672 ± 7	63.1 ± 1.2	12.7 ± 0.7

Table 2 shows that the signal buildup time in the energy transmission is slightly longer than that in the emission, with an opposite relationship apparent between the respective decay times. These results will be discussed below in detail.

**Figure 6:**
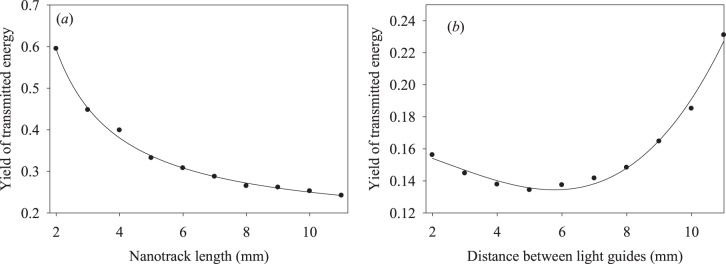
Efficiency of the energy transfer by the nanotrack (**a**) in function of its length, and (**b**) of the position of the output fiber optic cable on the 12 mm long nanotrack.

We also measured the transmitted energy yield in function of the nanotrack length and position of the fiber optic cable on the 12 mm long nanotrack, with its active length varying between 2 and 11 mm. The resulting plot is shown in Fig. 6. Note that the energy transfer yield decreases with the nanotrack length, while the energy transfer yield to the output fiber optic cable has a broad minimum. We shall discuss these results in more detail below.

### Dynamics of the energy transfer from the nanotrack to oxazine-170 dye

**Figure 7:**
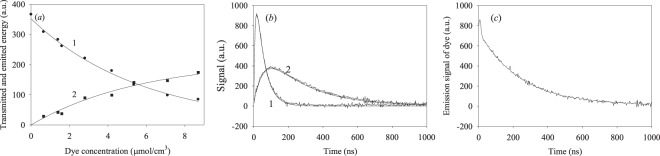
(**a**) Quantum yields of the energy (1) transmitted along the nanotrack and (2) emitted by the dye adsorbed at the nanotrack surface; (**b**) time evolution (1) of the energy transmitted along nanotrack and (2) radiation emitted by the dye; (**c**) emission of bulk oxazine-170. The excitation was performed at 633 nm and 5 mW (steady-state mode) or 630 nm and 5 mJ (pulsed mode).

We explored dynamics of the energy transfer from the nanotrack excited at 630 nm, by immersing the nanotrack into a cell filled with oxazine-170 solutions at different concentrations. The quantum yields of energy transfer *ϕ*_*t*_ and energy emission by the dye *ϕ*_*d*_ were measured in function of the dye concentration, with the results plotted in Fig. 7.

It follows from Fig. 7 that the quantum yield of energy transfer along the nanotrack decreases with the growth of oxazine-170 concentration in methanolic solutions, while the quantum yield of adsorbed dye emission increases. These results directly demonstrate the energy transfer from the nanotrack to the adsorbed dye molecules. To better understand the energy transfer dynamics from the excited nanotrack to the adsorbed dye molecules, we performed time-resolved experiments on the energy transfer and dye emission dynamics. Typical kinetics are shown in Fig. 7b; these were fitted using the Eq. (2), where we are only interested in the *τ*_1_ and *τ*_2_ lifetimes denoted here as *τ′*_1_ and *τ′*_2_. Fitting parameters are listed in Table 3.

**Table 3:** Fitting parameters for the emission dynamics in presence of dye obtained using Eq. (2).

Parameter	*τ*_1_′, ns (decay)	*τ*_2_′, ns (buildup)
Energy transmitted to the second fiber-optic cable	47.2 ± 1.2	9.1 ± 0.7
Energy emitted by the dye	239.2 ± 4.2	53.3 ± 1.3

The respective fitting parameters are listed in Table 3. Note that the dye emission decay time is in good agreement with the results obtained upon direct excitation (241 ns; see Fig. 6c). As we already noted, we demonstrated efficient energy transport from the excited nanotrack to the adsorbed dye molecules, and next we shall discuss the energy transfer mechanism.

## Data Analysis

### Energy transmission along the nanotrack

Data shown in Figs 2–5 will be discussed within the modeling approach developed earlier^16–18^, where it was shown that the spectra of Fig. 2 may be interpreted using the expression for the electronic energy levels in a potential box with infinite walls^26^. Thus, the spectral maxima will be described by the relationship

3 $${\nu }_{n,n+1}[eV]=0.3749\frac{1}{f{a}^{2}}[2mn+{m}^{2}]$$

where *f* = *m*_*eff*_*/m*_*e*_, *m*_*eff*_ is the effective electron mass, *m*_*e*_ is the free electron mass, *a* is the nanolayer thickness, *n* is the quantum number of the highest populated level, *n* + *m* is the quantum number of the excited state, and *m* is the quantum number increment. Figure 3 shows the experimental band maxima (Fig. 2a and b) in function of the quantum number increment, fitted with the relationship (3). We already noted that *f* and *n* are 0.17123 and 9, respectively, in good agreement with the values reported earlier^17–22^. Figure 2b shows interesting results, presenting the energy transfer efficiency in function of the probing radiation wavelength. Indeed, the absorption minima in Fig. 2a correspond to the maxima of the energy transfer efficiency in Fig. 2b.

The data shown in Fig. 4 demonstrate the reduction in both the energy transfer efficiency and the nanotrack emission. This may be explained as saturation, where the energy transfer efficiency by a two-level system (with the ground state, and one excited state) is given by

4 $$\eta =\frac{C{k}_{t}}{{W}_{abs}+{k}_{t}+{k}_{nr}+{k}_{se}}.$$

Here *C* is a constant, *W*_*abs*_ is the photon absorption rate by the nanotrack, *k*_*t*_ is the energy transfer rate constant along the nanotrack, *k*_*nr*_ is the radiationless relaxation rate constant and *k*_*se*_ the rate constant of the nanotrack emission. We used Eq. (4) to fit the data of Fig. 4, with the average ${k}_{t}+{k}_{nr}+{k}_{se}=(3.2\pm 0.7)\times {10}^{7}\,{s}^{-1}$.

The results of time-resolved measurements are shown in Fig. 5, with the respective fitting parameters listed in Table 2.

**Figure 8:**
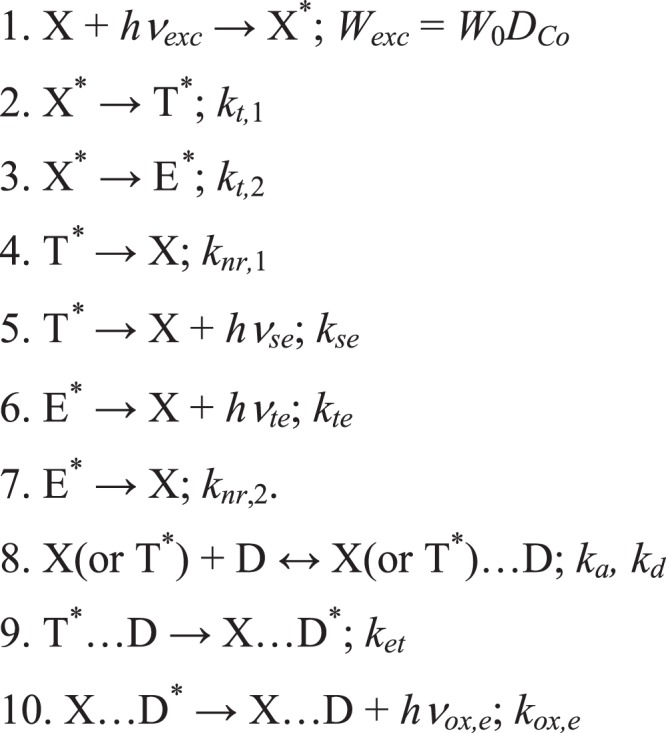
Kinetic scheme of the excited state dynamics in Co nanotrack and Co nanotrack with adsorbed oxazin dye.

We interpret these results using a phenomenological model, detailed in Fig. 8. It describes the energy transfer along the nanotrack (1–7) and to the adsorbed dye (9–10); reaction (8) describes the adsorption/desorption equilibrium of the dye D (oxazin-170), independent on the excitation of the nanotrack. X and X* are the ground and the electronic excited states of the nanotrack, the latter generated by light coming through the first light guide. We identify T* states as surface plasmons, which get distributed homogeneously over the nanotrack. We identify E* states as excitons; these are probed in the measurement zone by the second light guide (Fig. 1a). The radiationless relaxation constant *k*_*nr*,2_ of E* states depends on the distance from the excitation zone (Fig. 1a). *W*_0_ is the excitation source intensity and *D*_*Co*_ is the absorption optical density of the Co nanotrack at 633 nm (see Fig. 2) in steady state experiments, the respective expression is valid at low *D*_*Co*_ values; in pulsed experiments the energy absorbed by the nanotrack at 630 nm is proportional to the pulse energy, and is given at low *D*_*Co*_ values by:

$$W={D}_{Co}{\int }_{0}^{\infty }{W}_{0}(t)dt.$$

Here we introduced separate T* and E* excited states in order to distinguish between the integral emission from the nanotrack surface, and the emission originating in the zone probed by the second optical cable. Such separation may be justified by the difference in the respective boundary conditions. We shall leave the detailed analysis of the energy transfer mechanism along the nanotrack for a follow-up paper.

The intensities of the surface emission (T* states) and the transferred emission (E* states) are given by:

5 $${I}_{s}(t)=\frac{{k}_{se}{k}_{t,1}{[{X}^{\ast }]}_{0}}{({k}_{t,1}+{k}_{t,2})-({k}_{nr,1}+{k}_{se})}({e}^{-({k}_{nr,1}+{k}_{se})t}-{e}^{-({k}_{t,1}+{k}_{t,2})t})$$

6 $${I}_{t}(t)=\frac{{k}_{te}{k}_{t,2}{[{X}^{\ast }]}_{0}}{({k}_{t,1}+{k}_{t,2})-({k}_{te}+{k}_{nr,2})}({e}^{-({k}_{te}+{k}_{nr,2})t}-{e}^{-({k}_{t,1}+{k}_{t,2})t})$$

where [X*]_0_ is the initial excitation density in the nanotrack. Comparing relationships (5) and (6) with the parameters of Eq. (2), we identify the parameters of Table 2 as follows: (*τ*_1_)^−1^ = *k*_*nr*,1_ + *k*_*se*_ and (*τ*_2_)^−1^ = *k*_*t*,1_ + *k*_*t*,2_ for the surface emission, and (*τ*_1_)^−1^ = *k*_*te*_ + *k*_*nr*,2_ and (*τ*_2_)^−1^ = *k*_*t*,1_ + *k*_*t*,2_ for the transferred energy. Note that the two *τ*_2_ values should be equal in the phenomenological model proposed, as we in fact observe experimentally (Table 2), providing some justification for the model. Note also that the data of Fig. 6 are described quite well by the relationship (6), where we have to take into account that *k*_*nr*,1_ depends on the nanotrack length. Taking into account that the transmitted energy is defined as follows:

7 $${\phi }_{et}\approx \frac{{k}_{te}{k}_{t,2}}{({k}_{t,1}+{k}_{t,2})({k}_{te}+{k}_{nr,2})}\approx \frac{1}{[1+\xi (l)]}$$

we conclude that *k*_*nr*,2_ increases with the nanotrack length, where *k*_*t*,1_ < *k*_*t*,2_ and *ξ*(*l*) = *k*_*nr*,2_*/k*_*te*_. Thus, the length dependence of Fig. 6a may be described by Eq. (7), where assuming *ξ*(*l*) = *al*, we obtain *a* = 0.69 ± 0.07 *mm*^−1^. The linear dependence of *ξ*(*l*) is reasonable, as the effective radiationless relaxation efficiency of the nanotrack excited states (excitons) should be proportional to the effective exciton propagation time along the nanotrack. The results of Fig. 6b may be explained using the expression for the surface emission intensity Eq. (5) integrated over time and written as follows:

8 $$\begin{array}{l}{I}_{s}\approx \frac{{k}_{se}{k}_{t,1}{[{X}^{\ast }]}_{0}}{({k}_{t,1}+{k}_{t,2})({k}_{nr,1}+{k}_{se})}=u\frac{{k}_{se}}{({k}_{nr,1}+{k}_{se})}\\ u=\frac{{k}_{t,1}{[{X}^{\ast }]}_{0}}{({k}_{t,1}+{k}_{t,2})}\end{array}$$

where we assume that ${k}_{nr,1}=a^{\prime} +bl\approx a^{\prime}$, while *k*_*se*_ is a nonlinear function of *l* dependent on the nanotrack shape. Presently we use ${k}_{se}=a^{\prime\prime} +bl+c{l}^{2}$, with the fitting parameters *a*″*/a*′ = 0.21, *b/a*′ = −0.028 and *c/a*′ = 2.7 × 10^−3^. We shall discuss the detailed mechanism behind this phenomenological approach in a follow-up publication. Here, we only note that such mechanism uses the exciton-plasmon energy-transfer pathway. We shall discuss the first step of this mechanism below, leaving a more detailed treatment for a future study.

We shall now discuss the physical mechanism of energy transfer along the nanotrack. Let us first consider the electronic state resulting due to photon absorption. We assigned the band at 1.9639 eV to the *n* = 14 ← *n* = 9 transition between the discrete states generated by quantum confinement. However, we should take into account the diabatic interactions between these discrete states and the continuous spectrum describing the electron motion along the nanotrack surface. The discrete and continuous-state wavefunctions may be written as follows^26^:

9 $$\begin{array}{c}{\psi }_{d}=\sqrt{\frac{2}{a}}Sin(\frac{\pi n}{a}z)\\ {\psi }_{c}=\frac{1}{\sqrt{2\pi \hslash \sqrt{\frac{2E}{{m}_{e}}}}}({e}^{ikr}+{e}^{-ikr}){e}^{i\frac{E}{\hslash }t}\end{array}$$

where *m*_*e*_ *f* is the effective electron mass, and the *z* axis is normal to the nanotrack. Thus, the transition rate from the discrete state to the continuous state may be described by the golden Fermi rule, and presented as follows:

10 $${k}_{dc}=\frac{2\pi }{\hslash }{|\langle {\psi }_{d}|{\hat{V}}_{nd}|{\psi }_{c}\rangle |}^{2}{\rho }_{c}({E}_{d})$$

Here, ***V***_*nd*_ is the diabatic perturbation mixing the states of interest and *ρ*_*c*_(*E*_*d*_) is the state density in the continuous spectrum at the discrete excited state energy. We calculated this rate constant using a homemade FORTRAN code, where *ρ*_*c*_(*E*_*d*_) was calculated using the method discussed earlier^21^, with the resulting value *k*_*dc*_ = 5.74 × 10^7^ s^−1^. Note that this energy transfer into the continuous spectrum causes energy transport along the nanotrack with simultaneous weak emission from the track surface and a stronger emission at the end of track, probably due to the appropriate boundary conditions existing there. Apparently the energy propagation rate along the track is much higher than *k*_*dc*_, therefore the buildup time of the kinetics of Fig. 5 (Table 2, last column for *τ*_2_) should be interpreted as 1/*k*_*dc*_ ≈ 17.5 ns. Thus, we have as least a qualitative physical understanding of the experimental results provided by the physical mechanism proposed. Next we shall discuss the energy transfer from the nanotrack to the dye.

### Energy transfer from the nanotrack to oxazine-170

The data of Fig. 7 present novel information, directly demonstrating the energy transfer dynamics from the excited nanotrack to oxazine-170 dye adsorbed at its surface. In this case, our phenomenological mechanism (1–7, Fig. 8) should be complemented by the reactions (8–10), where X…D is the dye adsorbed at the nanotrack surface, T*…D is the excited Co nanotrack with adsorbed oxazine, X…D* are the excited oxazine-170 molecules adsorbed by the nanotrack. The transferred energy is described by Eq. (6), while the energy emitted by the adsorbed dye may be obtained in the form:

11 $${I}_{ox}(t)\approx \frac{{k}_{ox,e}{k}_{et}{k}_{t,1}{[X{}^{\ast }]}_{0}}{[({k}_{t,1}+{k}_{t,2})-({k}_{nr,1}+{k}_{se}+{k}_{et})]({k}_{nr,1}+{k}_{se}+{k}_{et})}({e}^{-{k}_{ox,e}t}-{e}^{-({k}_{nr,1}+{k}_{se}+{k}_{et})t})$$

Comparing the latter expression with the expression fitted to the dye emission kinetics, we identify ${k}_{ox,e}={(\tau {^{\prime} }_{2})}^{-1}$ and $({k}_{nr,1}+{k}_{se}+{k}_{et})={(\tau {^{\prime} }_{1})}^{-1}$. These results seem quite reasonable taking into account the experimental values of *τ*_1_, *τ*_2_, *τ*′_1_ and *τ*′_2_.

Our phenomenological model does not consider either the dye adsorption dynamics or the nature of the *k*_*et*_ rate constant of the energy transfer from the nanotrack to the dye. We shall consider these two issues below.

#### Adsorption-desorption dynamics

The dye adsorption dynamics may contribute to the results of Fig. 6a. The dye adsorption rate constant may be written in the form^34^:

12 $$\begin{array}{c}{k}_{a}={\gamma }_{a}\frac{{S}_{nt}}{{V}_{eff}}{\bar{\upsilon }}_{ox}={\gamma }_{a}\frac{{D}_{ox}}{{h}^{2}}\\ {\bar{\upsilon }}_{ox}=\frac{{D}_{ox}}{h};{V}_{eff}={S}_{nt}h\end{array}$$

where *γ*_*ax*_ is the efficiency of dye absorption by nanotrack surface, *S*_*nt*_ is the nanotrack surface area, *V*_*eff*_ is the effective volume of the sample cell $({V}_{eff}={S}_{nt}h)$, ***υ***_*ox*_ is the average diffusion velocity of dye molecules in methanol, *D*_*ox*_ is the dye diffusion coefficient in methanol, *h* is the effective adsorption distance between the dye and the nanotrack. We assumed that *γ*_*ax*_ = 1, obtaining the adsorption rate in the form:

13 $${W}_{a}=\frac{{D}_{ox}}{{h}^{2}}[Ox]$$

The dye desorption process may be described by a monomolecular rate constant, written as follows^34^:

14 $${k}_{d}={A}_{d}{e}^{-\frac{{E}_{ac}}{{k}_{B}T}}$$

where *A*_*d*_ is the preexponential factor and *E*_*ac*_ is the activation energy. We estimate these parameters considering oxazine (MW = 431.57) as a structureless spherical particle, linked to the nanotrack by van der Waals interactions with the interaction energy of ca. 0.25 eV^35^. To simplify the analysis, the Lennard-Jones 6–12 potential was approximated by the Morse potential^36^:

15 $${U}_{M}(z)={E}_{ac}{(1-{e}^{-\alpha (z-{z}_{0})})}^{2}$$

where *E*_*ac*_ = 0.25 eV, *α* defines the potential width and *z*_0_ is where the potential minimum occurs (the *z* axis is perpendicular to the nanotrack). In this case, *A*_*d*_ depends on the vibrational frequency of oxazine along the *z* axis, with the elasticity constant given by^37^:

16 $$\kappa ={\frac{{\partial }^{2}{U}_{M}(z)}{\partial {z}^{2}}|}_{z={z}_{0}}=2{\alpha }^{2}{E}_{ac}$$

Using the typical van der Waals bond length of 5–7 Å^37^, we assume α = 0.2 Å^−1^. Thus, we obtain for *A*_*d*_^35^:

17 $${A}_{d}=\sqrt{\frac{\kappa }{{M}_{ox}}};\,[c{m}^{-1}]$$

The estimate produced *A*_*d*_ = 0.73 cm^−1^, corresponding to the desorption rate constant value of ca. 1.6 × 10^5^ s^−1^. Thus, the concentration of the adsorbed oxazine dye may be estimated using the equilibrium reaction (9) on Fig. 8.

Thus, we may write

18 $$[X\mathrm{...}D]\approx \frac{{D}_{ox}}{{k}_{d}h}{[Ox]}_{0},c{m}^{-2}\,$$

The latter relationship is correct only if *S*_*nt*_*/V*_*eff*_ ≫ 1, thus an acceptable value of *h* is about 10 Å^32^. In this case, the relation (18) may be rewritten as follows:

19 $$[X\ldots D]\approx \frac{{D}_{ox}}{{k}_{d}h}{[Ox]}_{0}\approx 1.3\times {10}^{-2}\,\mu mol/c{m}^{2}$$

Thus, the above-mentioned conditions correspond to high surface concentrations of the dye. Therefore, we conclude that the amount of adsorbed dye is proportional to the dye concentration in solution, thus the data of Fig. 7a may be explained using Eq. (19) and the time-integrated Eq. (11). Combining these time-integrated dependences with Eq. (19), we obtain proportionality with the surface concentration of the adsorbed dye. We shall next discuss the origins of the *k*_*et*_ rate constant.

#### Nature of the *k*_et_

We may consider two mechanisms for the energy transfer from the excited Co nanotrack to the adsorbed dye molecules: (1) contact exchange mechanism^38^ and (2) electric dipole-dipole mechanism^39^.

1. The contact exchange mechanism is induced by exchange interaction^38^.
  We consider two-electron exchange interaction described by:
  20 $${\hat{V}}_{exch}=\frac{1}{4\pi \varepsilon {\varepsilon }_{0}}\frac{{e}^{2}}{|{z}_{1}-{z}_{2}|}$$
  The energy transfer may be described by the golden Fermi rule, Eq. (10), where the matrix element of the exchange interaction is given by:
  21 $$\langle {V}_{exch}\rangle =\frac{1}{4\pi \varepsilon {\varepsilon }_{0}}\langle {\psi }_{d}^{\ast }{\psi }_{ox}|\frac{{e}^{2}}{|{z}_{1}-{z}_{2}|}|{\psi }_{d}{\psi }_{ox}^{\ast }\rangle $$
  where *ψ*_d_* and *ψ*_*d*_ are given by the first line of Eq. (9), using respectively *n* = 15 and *n* = 9, *ψ*_*ox*_ and *ψ*_ox_* are the wave functions of the ground and excited state of oxazine-170. We used the structure of oxazine-170^40^ to calculate its *ab initio* electronic ground and excited state wavefunctions and the energy gap between them. These calculations used Gaussian-2000 software package, and the coupled clusters method with the 6–31 G(d) basis set. The calculated energy gap between the states of interest is about 1.89 eV, in good agreement with the experimental value of 1.87 eV^40,41^. Therefore, such calculations should be also producing reasonable wavefunctions. Thus, we used the output of the Gaussian-2000 package in our FORTRAN code that calculated the matrix element of Eq. (21), using *z*_0_ = 5 Å (see above) as the average distance between the center of mass of the oxazine molecule and the nanotrack. We calculated the state density of oxazine at the excited-state energy of the nanotrack as follows^39^:
  22 $${\rho }_{c}({E}_{d})=\frac{{({E}_{d,ox}+a\sum _{i}\hslash {\omega }_{i})}^{s}}{(s-1)!\prod _{i}\hslash {\omega }_{i}}$$
  where *E*_*d,ox*_ = 0.09 eV is the energy gap between the excited nanotrack and excited oxazine, *ω*_*i*_ is the frequency of the fundamental mode of dye molecule in the excited state (calculated *ab initio*), *a* = 0.5, and *s* = 150 is the number of vibrational degrees of freedom for the C_21_H_22_N_3_O_5_Cl molecule. The calculations produced *k*_*et*_ = 1.07 × 10^7^ s^−1^.
2. Electric dipole-dipole interaction.

Reproducing the deductions of the previous section, we reduce the problem to calculating the electronic matrix element

23 $${V}_{DA,dd}=\langle {\psi }_{d}^{\ast }{\psi }_{ox}|{\hat{V}}_{dd}|{\psi }_{d}{\psi }_{ox}^{\ast }\rangle $$

with

24 $${\hat{V}}_{dd}=\frac{{e}^{2}}{\varepsilon {\varepsilon }_{0}\cdot {r}^{3}}[({\overrightarrow{r}}_{d}\cdot {\overrightarrow{r}}_{ox})-\frac{3}{{r}^{2}}({\overrightarrow{r}}_{d}\cdot \overrightarrow{r})({\overrightarrow{r}}_{ox}\cdot \overrightarrow{r})]$$

Here, *r* is the distance between the oscillator centers, *r*_*d*_ and *r*_*ox*_ distances between the centers of the oscillators and the optical electrons of *Co* nanotrack and oxazine molecule, respectively, according to Förster^39^. For the model in analysis, we rewrite the latter relationship as:

25 $${\hat{V}}_{dd}=\frac{{e}^{2}}{\varepsilon \cdot {r}^{3}}({\overrightarrow{r}}_{d}\cdot {\overrightarrow{r}}_{ox})[1-3Co{s}^{2}(\theta )]$$

where *θ* is the angle between the *z* axis and the ***r*** vector. Averaging over the angle, we obtain for the interaction:

26 $${\hat{V}}_{DA,dd}=-\frac{{e}^{2}}{\pi \varepsilon \cdot {r}^{3}}({\overrightarrow{r}}_{d}\cdot {\overrightarrow{r}}_{ox})$$

We therefore obtain for the dipole-dipole interaction:

27 $${V}_{DA,dd}^{el}=-\frac{{e}^{2}}{\pi \varepsilon \cdot {r}^{3}}\langle {\psi }_{d}^{\ast }{\psi }_{ox}|({\overrightarrow{r}}_{d}\cdot {\overrightarrow{r}}_{ox})|{\psi }_{d}{\psi }_{ox}^{\ast }\rangle $$

The calculated dipole-dipole interaction rate constant is *k*_*et*_ = 1.72 × 10^6^ s^−1^. Therefore, taking into account both contributions, we obtain *k*_*et*_ = 1.24 × 10^7^ s^−1^, with the contact exchange interaction providing the main contribution. We shall discuss these results in the following section. We shall leave for the next report the more precise a*b initio* calculations that take dye-surface interactions into account.

## Discussion

The observed macroscopic-scale energy propagation in the metal film is an interesting quantum effect, studied here for a Co nanotrack. However, such phenomena were extensively studied and discussed earlier^42–44^, and analyzed using the quasi-classic plasmon/polaron theoretical approach. The difference between the plasmon/polaron effect and the presently discussed effects is in the nature of the electronic oscillations in the physical system studied^24^. Plasmon/polaron is a coherent longitudinal oscillation of the electron ensemble at the metal surface, while the presently studied excitations, similar to excitons^24^, are interpreted as a coherent transverse oscillation of the electron ensemble occurring along the QC coordinate, i.e. perpendicular to the metal surface^24^. Note, however, that the coherent wave-package of the transverse electronic oscillations created within a certain zone of the metal film may induce similar electronic oscillations in the neighboring zone^24^, the mechanism that we shall leave for a follow-up publication. Presently we considered the excitation propagation mechanism that includes exciton-plasmon transformation dynamics, and further plasmon propagation along the Co nanotrack. Note that the nanotrack configuration significantly affects the energy transfer efficiency. We shall devote a follow-up study to the shape optimization and theoretical analysis of the detailed mechanism of exciton-plasmon and exciton-exciton energy transfer.

We also explored the energy transfer from the excited Co nanotrack (energy donor) to adsorbed dye molecules (energy acceptor). We believe these results are very promising for future applications in optoelectronics, optical communications, and biomedical research, as we are able to attach any suitable energy acceptor(s) at any point of the nanotrack, allowing to supply energy to these acceptors in a pre-programmed sequence, which would allow e.g. to perform a series of biochemical reactions in a nano-sized reactor. As the absorption spectrum of the nanotrack is dependent on its thickness, we are also able to produce a film that would have its energy levels in resonance with the energy gap between triplet and singlet states of the oxygen molecule, opening the path to photodynamic surface oxidation processes for destroying chemical or biological contaminants in air or water. Note that the estimated energy transfer rate constant from the nanotrack to the adsorbed dye is about 1.24 × 10^7^ s^−1^, demonstrating very efficient energy transfer between the energy donor and the energy acceptor, limited only by the lifetime of the excited Co nanotrack. The presently reported results are entirely novel, as we failed to find any publications reporting anything similar.

Coming back to the estimated dynamic parameters of the energy propagation along the nanotrack, the typical energy propagation time along 1.1 cm distance is 12.9 ns, corresponding to the energy transfer speed of 8 × 10^7^ cm/s. These values are quite low. Indeed, according to earlier reports^42–44^, the plasmon/polaron propagation speed along a metal surface is about 1.4 × 10^10^ cm/s. The low energy transfer speeds observed in our experiments result from low energy transfer rates from the discrete energy spectrum (transverse electronic oscillations) to the continuous energy spectrum (longitudinal electronic oscillations), much lower than the surface plasmon propagation velocity. The characteristic energy transfer time between the two spectra is about 17.4 ns, in reasonable agreement with the energy propagation velocity along the Co nanotrack (1.1 cm/17.4 ns ≈ 6.3 × 10^7^ cm/s). This explanation agrees with the earlier reports^45^ that found that free-space propagating waves and surface waves may be coupled by a gradient negative-permittivity material and the coupling may be enhanced if the material permittivity variation is suitably designed. On the other hand, it was also shown that excitation and propagation of surface plasmons have different time scales. However, presently we have not explored the possibility of the exciton (transverse coherent vibration) propagating along the nanotrack all by itself, leaving this mechanism for a follow-up study.

### Limitations

We did not consider the detailed mechanism of the macro-scale exciton propagation along the metal nanotrack; also, the mechanism explaining the dependence shown in Fig. 6b was not discussed. Both mechanisms will be reported on later, along with an extended set of experimental data.

## Conclusion

Presently we reported macroscopic-scale propagation of the excited-state energy (excitons) along a metal nanotrack and the energy transfer from the nanotrack to the adsorbed dye molecules. We explored the dynamics of both processes experimentally, finding that the effective velocity of the excitation propagation along the nanotrack is ca. 8 × 10^7^ cm/s, much lower than the surface plasmon propagation speed of 1.4 × 10^10^ cm/s. Assuming that the energy transfer occurs between excitons and surface plasmons, with subsequent propagation of surface plasmons, we conclude that this energy transfer should be the limiting step for the energy propagation. We estimate that the rate constant of such energy transfer is ca. 5.7 × 10^7^ s^−1^, in support of our conclusions. The energy transfer between the nanotrack and the adsorbed dye molecules is limited by the excited state lifetime in the nanotrack. This conclusion is supported by the buildup time of the adsorbed dye emission being much longer than the characteristic time of the energy transfer, estimated at 81 ns. Indeed, this characteristic time is quite similar to the dye emission buildup time.
